# Draft genome sequence of multidrug-resistant *Escherichia coli* F14E isolated from a captive common peacock with colisepticemia

**DOI:** 10.1128/mra.00927-25

**Published:** 2025-10-07

**Authors:** Khaled Hassan Shuvo, Tima Tisa Mallick, Naim Siddique, Ziban Chandra Das, Abu Nasar M. Aminoor Rahman, M. Nazmul Hoque

**Affiliations:** 1 Molecular Biology and Bioinformatics Laboratory, Department of Gynecology, Obstetrics and Reproductive Health, Gazipur Agricultural University 198780, Gazipur, Bangladesh; University of Maryland School of Medicine, Baltimore, Maryland, USA

**Keywords:** draft genome, *E. coli*, colisepticemia, multidrug-resistance, virulence factors, peacock

## Abstract

We report the draft genome sequence of *Escherichia coli* F14E, a multidrug-resistant strain isolated from the fecal sample of a captive common peacock (*Pavo cristatus*) diagnosed withcolisepticemia. The assembled genome comprises approximately 4.7 Mb distributed across 130 contigs, with 50.50% GC content and 99.27% genome completeness.

## ANNOUNCEMENT

*Escherichia coli,* a gram-negative facultative anaerobe, comprises both commensal strains and pathogenic variants causing diseases in multiple hosts (1, 2). The rise of multidrug-resistant (MDR) *E. coli* strains is a global public health concern (3). Wildlife, including peacocks, can act as a reservoir, spreading antimicrobial resistance genes (ARGs) via environmental pathways (4, 5). Colisepticaemia, caused by *E. coli*, is a pathogenic condition that can occur in various avian species, including peacocks (6, 7). *E. coli* strains causing colisepticemia remain challenging to control and present zoonotic risks (8). Investigating avian-derived *E. coli* isolates enhances understanding of resistance–virulence dynamics (6). We report the isolation and genomic characterization of an MDR, pathogenic *E. coli* strain (F14E) from a colisepticaemic peacock.

Twelve freshly voided fecal samples were aseptically collected from a 21-month-old common peacock diagnosed with colisepticemia at the Wildlife Reproduction and Conservation Field Laboratory, Gazipur Agricultural University (24.09°N, 90.41°E), Bangladesh. Samples were collected using sterile stool tubes, diluted with sterile distilled water, vortexed, and preserved for subsequent analysis. For *E. coli* isolation, samples were enriched overnight in nutrient broth (Oxoid, UK) at 37°C under aerobic conditions, serially diluted (10^−3^), and streaked onto Eosin-methylene blue agar (Oxoid, UK). Metallic green sheen colonies observed after 24 h were sub-cultured, and the isolate was identified as *E. coli* based on morphology, Gram staining, and biochemical tests (5, 9). Species-level identification of *E. coli* was confirmed using the VITEK-2 system v9.01 (10). Strain F14E exhibited resistance to ampicillin, azithromycin, oxacillin, sulfonamides, and tetracycline, as determined by Kirby-Bauer disk diffusion following CLSI M100 (2023) guidelines (11). For DNA isolation, strain F14E was grown in nutrient broth at 37°C for 18–24 h under aerobic conditions. Genomic DNA was extracted with the QIAamp DNA Mini Kit (QIAGEN, Germany) and quantified using NanoDrop 2000 (Thermo Fisher, USA) (12). Libraries were prepared from 1 ng DNA using the Nextera DNA Flex Kit (Illumina, USA) according to the manufacturer’s protocol, which includes enzymatic fragmentation and size selection, and sequenced (2×250 bp) on an Illumina MiSeq platform. Raw reads (*N* = 5,023,404) were trimmed with Trimmomatic v0.39 (13) and assessed with FastQC v0.12.1 (14). Genome assembly was performed with SPAdes v4.2.0 (15) and annotated using the NCBI Prokaryotic Genome Annotation Pipeline v6.10 (16). Genome completeness, virulence factor genes, ARGs, metabolic pathways, and human pathogenic potential were evaluated using CheckM v1.2.3 (17, 18, 18, 19, 19), RAST server (20), and PathogenFinder2 v0.5.0 (21), respectively. Default parameters were used for all software unless otherwise specified.

The key genomic characteristics of *E. coli* F14E are presented in Table 1. The draft genome was assembled into 130 contigs, totaling approximately 4.67 Mbps with 50.5% GC content, and 99.27% genome completeness (0.38% contamination). We predicted 51 ARGs and 59 virulence factor genes (VFGs), along with 377 functional subsystems. Additionally, the strain was linked to 105 pathogenic families and exhibited a predicted score of 0.976 (out of 1.0) for being a human pathogen. These results highlight the potential role of *E. coli* F14E in colisepticemia in peacock, emphasizing its relevance in veterinary and public health research.

**TABLE 1:** General genomic features of the *E. coli* F14E isolated from the feces of a captive common peacock with colisepticemia

Genome features	*E. coli s*train F14E
Genome size (bp)	4,667,555
Genome coverage (×)	133 ×
Genome completeness (%)	99.27 (0.38% contamination)
No. of contigs	130
Contig N_50_ (bp)	106,961
GC content (%)	50.5
Coding sequences (CDSs)	4,544
Protein-coding genes	4,345
No. of subsystems	377
No. of VFGs	59
No. of ARGs	51
PathogenFinder2 score	0.976
Genome accessions	JBOEQB000000000
Sequence read archive (SRA) accessions	SRR33697068

## Data Availability

The whole-genome shotgun sequencing project of the *E. coli* strain has been deposited in GenBank and the NCBI Sequence Read Archive (SRA) under BioProject accession number PRJNA1267841. The SRA accession is listed in Table 1. The version described in this paper is version JBOEQB000000000.1.

## References

[1] Kaper JB, Nataro JP, Mobley HL. 2004. Pathogenic Escherichia coli. Nat Rev Microbiol 2:123–140. doi:10.1038/nrmicro818 15040260

[2] Arafat KY, Hasnat S, Siddique N, Rahman MM, Homa SF, Hoque MN. 2025. Draft genome sequencing of a multidrug-resistant and virulent Escherichia coli strain isolated from milk of a cow with clinical mastitis. Microbiol Resour Announc 14:e0126024. doi:10.1128/mra.01260-24 39964251 PMC11895442

[3] Pitout JDD, Laupland KB. 2008. Extended-spectrum beta-lactamase-producing Enterobacteriaceae: an emerging public-health concern. Lancet Infect Dis 8:159–166. doi:10.1016/S1473-3099(08)70041-0 18291338

[4] Allen HK, Donato J, Wang HH, Cloud-Hansen KA, Davies J, Handelsman J. 2010. Call of the wild: antibiotic resistance genes in natural environments. Nat Rev Microbiol 8:251–259. doi:10.1038/nrmicro2312 20190823

[5] Hoque MN, Faisal GM, Jerin S, Moyna Z, Islam MA, Talukder AK, Alam MS, Das ZC, Isalm T, Hossain MA, Rahman A. 2024. Unveiling distinct genetic features in multidrug-resistant Escherichia coli isolated from mammary tissue and gut of mastitis induced mice. Heliyon 10:e26723. doi:10.1016/j.heliyon.2024.e26723 38434354 PMC10904246

[6] Barbieri NL, Tejkowski TM, de Oliveira AL, de Brito BG, Horn F. 2012. Characterization of extraintestinal Escherichia coli isolated from a peacock (Pavo cristatus) with colisepticemia. Avian Dis 56:436–440. doi:10.1637/9921-090811-Case.1 22856209

[7] Saha O, Hoque MN, Islam OK, Rahaman MM, Sultana M, Hossain MA. 2020. Multidrug-resistant avian pathogenic Escherichia coli strains and association of their virulence genes in Bangladesh. Microorganisms 8:1135. doi:10.3390/microorganisms8081135 32727140 PMC7465658

[8] Ievy S, Hoque MN, Islam MS, Sobur MA, Ballah FM, Rahman MS, Rahman MB, Hassan J, Khan MFR, Rahman MT. 2022. Genomic characteristics, virulence, and antimicrobial resistance in avian pathogenic Escherichia coli MTR_BAU02 strain isolated from layer farm in Bangladesh. J Glob Antimicrob Resist 30:155–162. doi:10.1016/j.jgar.2022.06.001 35671989

[9] Hoque MN, Istiaq A, Clement RA, Gibson KM, Saha O, Islam OK, Abir RA, Sultana M, Siddiki AZ, Crandall KA, Hossain MA. 2020. Insights into the resistome of bovine clinical mastitis microbiome, a key factor in disease complication. Front Microbiol 11:860. doi:10.3389/fmicb.2020.00860 32582039 PMC7283587

[10] Kumaran D, Laflamme C, Ramirez-Arcos S. 2023. A multiphasic approach to solve misidentification of Cutibacterium acnes as Atopobium vaginae during routine bacterial screening of platelet concentrates using the VITEK 2 system. Access Microbiol 5:000539. doi:10.1099/acmi.0.000539.v3 PMC10323807 37424557

[11] Rai S, Dash D, Agarwal N. 2023. Introducing the new face of CLSI M100 in 2023: an explanatory review. Indian J Med Microbiol 46:100432. doi:10.1016/j.ijmmb.2023.100432 37945125

[12] García-Alegría AM, Anduro-Corona I, Pérez-Martínez CJ, Guadalupe Corella-Madueño MA, Rascón-Durán ML, Astiazaran-Garcia H. 2020. Quantification of DNA through the NanoDrop spectrophotometer: methodological validation using standard reference material and Sprague Dawley rat and human DNA. Int J Anal Chem 2020:8896738. doi:10.1155/2020/8896738 33312204 PMC7719535

[13] Bolger AM, Lohse M, Usadel B. 2014. Trimmomatic: a flexible trimmer for Illumina sequence data. Bioinformatics 30:2114–2120. doi:10.1093/bioinformatics/btu170 24695404 PMC4103590

[14] Lo C-C, Chain PSG. 2014. Rapid evaluation and quality control of next generation sequencing data with FaQCs. BMC Bioinformatics 15:366. doi:10.1186/s12859-014-0366-2 25408143 PMC4246454

[15] Bankevich A, Nurk S, Antipov D, Gurevich AA, Dvorkin M, Kulikov AS, Lesin VM, Nikolenko SI, Pham S, Prjibelski AD, Pyshkin AV, Sirotkin AV, Vyahhi N, Tesler G, Alekseyev MA, Pevzner PA. 2012. SPAdes: a new genome assembly algorithm and its applications to single-cell sequencing. J Comput Biol 19:455–477. doi:10.1089/cmb.2012.0021 22506599 PMC3342519

[16] Tatusova T, DiCuccio M, Badretdin A, Chetvernin V, Nawrocki EP, Zaslavsky L, Lomsadze A, Pruitt KD, Borodovsky M, Ostell J. 2016. NCBI prokaryotic genome annotation pipeline. Nucleic Acids Res 44:6614–6624. doi:10.1093/nar/gkw569 27342282 PMC5001611

[17] Parks DH, Imelfort M, Skennerton CT, Hugenholtz P, Tyson GW. 2015. CheckM: assessing the quality of microbial genomes recovered from isolates, single cells, and metagenomes. Genome Res 25:1043–1055. doi:10.1101/gr.186072.114 25977477 PMC4484387

[18] Zhou S, Liu B, Zheng D, Chen L, Yang J. 2025. VFDB 2025: an integrated resource for exploring anti-virulence compounds. Nucleic Acids Res 53:D871–D877. doi:10.1093/nar/gkae968 39470738 PMC11701737

[19] Alcock BP, Huynh W, Chalil R, Smith KW, Raphenya AR, Wlodarski MA, Edalatmand A, Petkau A, Syed SA, Tsang KK, et al.. 2023. CARD 2023: expanded curation, support for machine learning, and resistome prediction at the Comprehensive Antibiotic Resistance Database. Nucleic Acids Res 51:D690–D699. doi:10.1093/nar/gkac920 36263822 PMC9825576

[20] Overbeek R, Olson R, Pusch GD, Olsen GJ, Davis JJ, Disz T, Edwards RA, Gerdes S, Parrello B, Shukla M, Vonstein V, Wattam AR, Xia F, Stevens R. 2014. The SEED and the rapid annotation of microbial genomes using subsystems technology (RAST). Nucleic Acids Res 42:D206–D214. doi:10.1093/nar/gkt1226 24293654 PMC3965101

[21] Cosentino S, Voldby Larsen M, Møller Aarestrup F, Lund O. 2013. PathogenFinder--distinguishing friend from foe using bacterial whole genome sequence data. PLoS One 8:e77302. doi:10.1371/journal.pone.0077302 24204795 PMC3810466

